# Snapping hip caused by a venous hemangioma of the gluteus maximus muscle: a case report

**DOI:** 10.1186/1752-1947-2-386

**Published:** 2008-12-16

**Authors:** Cheng-Li Lin, Ming-Tung Huang, Chii-Jeng Lin

**Affiliations:** 1 Department of Orthopedics, National Cheng Kung University Medical Center, Tainan, Taiwan

## Abstract

**Introduction:**

Snapping hip, or coxa saltans, is defined as a clinical condition where a usually painful, audible snap occurs during hip flexion and extension. Its causes can be divided into external, internal or intra-articular origin. Accurate diagnosis is a prerequisite to successful treatment. We report a rare cause of snapping hip which is different from any previously reported cases.

**Case presentation:**

A 23-year-old man presented to us with right hip pain of more than 10 years duration. Atrophy of the right gluteus maximus with snapping and tenderness were also noted. The imaging study revealed a focal intramuscular lesion in the lateral portion of the right gluteus maximus muscle. Surgery was performed and pathological examination concluded this mass to be a venous hemangioma.

**Conclusion:**

Intramuscular hemangioma, though rare, should be considered in the differential diagnosis of a snapping hip even though muscle fibrosis is most frequently encountered.

## Introduction

A snapping hip, or coxa saltans, is characterized by a usually painful snapping phenomenon during hip flexion and extension. The causes of a snapping hip can be grouped into external, internal or intra-articular origin [1]. Since each cause represents a distinct pathogenesis and may consequently require different surgical interventions, accurate diagnosis is a prerequisite to successful treatment [2-7].

We report a relatively rare cause of a snapping hip. The etiology of the snapping hip of this 23-year-old man had been misinterpreted as muscle fibrosis, the most common cause for this symptom, for more than 10 years. Interestingly, the preoperative assessment and pathological findings were subsequently demonstrated to be different from any previously reported causes. The cause turned out to be an intramuscular tumor located close to the gluteus maximus insertion around the greater trochanter.

## Case presentation

A 23-year-old Taiwanese man, who had suffered from right hip pain for more than 10 years, requested an orthopedic consultation. He stated that a computed tomography (CT) examination 10 years ago for hip pain disclosed no abnormal findings. However, the hip pain persisted and radiated to the buttock intermittently, especially when he adducted and flexed his right hip joint. The pain could be temporarily relieved by either abduction or the use of painkiller medication.

**Figure 1:**
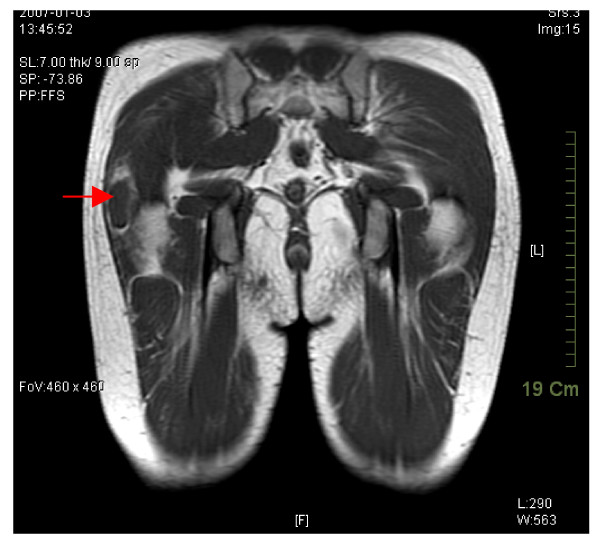
Magnetic resonance imaging study revealing a focal lesion within the lateral portion of the gluteus maximus muscle. Hypointense signal in T1W is seen under contrast enhancement.

**Figure 2:**
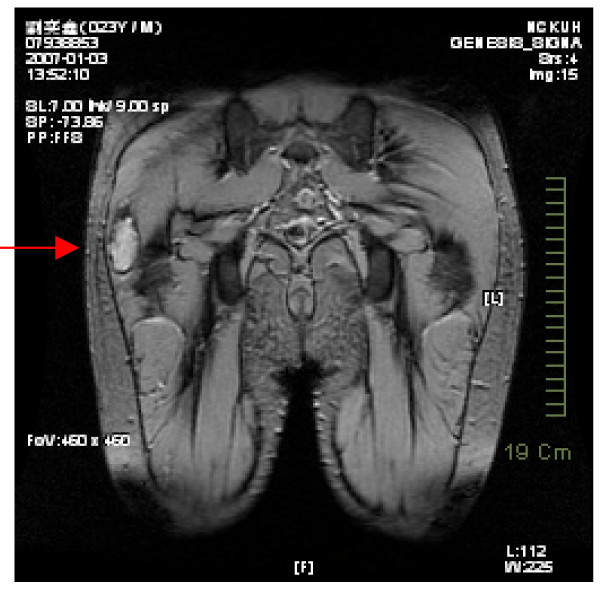
Hypointense signal in T1W under contrast enhancement.

On physical examination, atrophy of the right gluteus maximus with snapping and tenderness of the right hip was noted. A tight tensor fascia lata without an obvious palpable mass was suggested. Under the suspicion that this was not a case of common tensor fascia lata fibrosis, magnetic resonance imaging (MRI) was arranged and surprisingly revealed a focal intramuscular lesion in the lateral portion of the right gluteus maximus muscle, approximately 3.5 × 1.5 × 4.4 cm in size, which was hypointense in T1W and hyperintense in T2W under contrast enhancement (Figures 1 and 2).

**Figure 3:**
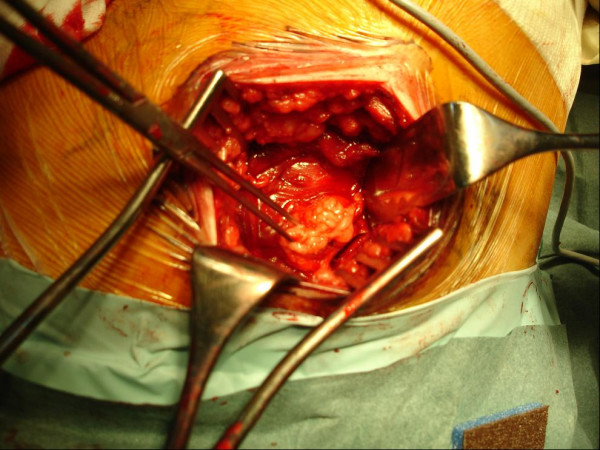
Operative findings. The soft tissue mass within the gluteus maximus muscle.

**Figure 4:**
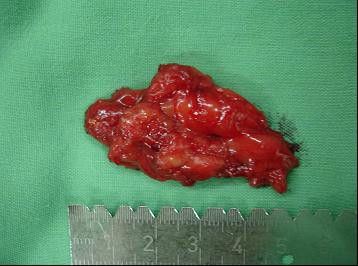
Specimen.

**Figure 5:**
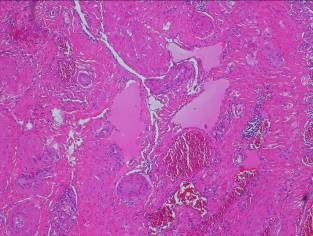
Pathology.

The patient agreed to surgical management for both the relief of the symptoms and to determine the pathology of the lesion. The gluteus maximus muscle was exposed during the operation and a hypervascular soft-tissue mass was noted (Figure 3). We performed surgical excision of the tumor (Figure 4) and pathological examination concluded this mass to be a venous hemangioma (Figure 5). The snapping phenomenon was no longer present after the surgery.

## Discussion

The most common type of snapping hip is the external type caused by the iliotibial band sliding over the greater trochanter [1,7]. The iliotibial band remains posterior to the greater trochanter when the hip is extended. As the hip moves into flexion, the iliotibial band, with assistance from the underlying bursae, glides smoothly over the greater trochanter to the anterior position. However, when the posterior portion of the iliotibial band or the anterior border with the gluteus maximus becomes thickened, this results in snapping of the tendon over the greater trochanter [8,9]. In such a case, Z-plasty is the most common surgical procedure for correcting an external snapping hip [2,3].

A tumor in the gluteus maximus muscle might mechanically irritate the smooth movement between the iliotibial band and the greater trochanter, or cause a tight tensor fascia lata, mimicking a snapping hip. However, very few cases have been reported concerning the relationship between a local tumor, not of intramuscular origin, and a snapping hip, except in 2005 when Sanshiro *et al*. reported a patient with external snapping hip caused by an osteochondroma of the proximal femur [10]. The symptoms disappeared after surgical resection of the osteochondroma.

In our patient, the hemangioma grew in the lateral part of the gluteus maximus muscle insertion superficial to the greater trochanter and disturbed the smooth movement of the iliotibial band over the greater trochanter. Such an etiology has not been reported before in the literature.

Skeletal muscle hemangioma is probably the most common form of hemangioma of deep soft tissue, and might be located in the retroperitoneum, mesentery or muscles of the lower extremities. Nevertheless, it is rare when considering the spectrum of benign vascular neoplasms [11]. It accounts for only 0.8% of all hemangiomas and, when it does occur, it affects the lower extremity most often [12].

According to histological subtyping, haemangiomas can be divided into capillary, cavernous and venous types. A venous hemangioma is rare and is characteristically present during adult life [13]. Pain is the cardinal symptom occurring in 60% of cases [14]. In our patient, a snapping hip was an extraordinarily rare presentation of a venous haemangioma. The crucial image modality is MRI while the choice of treatment is surgical excision [12].

## Conclusion

An intramuscular hemangioma, though rare, should be considered in the differential diagnosis of a snapping hip even though muscle fibrosis is the most frequent cause.

## Consent

Written informed consent was obtained from the patient for publication of this case report and any accompanying images. A copy of the written consent is available for review by the Editor-in-Chief of this journal.

## Competing interests

The authors declare that they have no competing interests.

## Authors' contributions

CLL collected the patient data, reviewed the literature and was a major contributor in writing the manuscript. MTH advised about the writing of the article, interpreted the clinical significance of the aetiology and participated in the surgical intervention. CJL was the visiting staff member for the patient and was a major contributor in writing the manuscript. All authors read and approved the final manuscript.
